# Medical Society of London

**Published:** 1853-02

**Authors:** 


### RECORD OF MEDICAL SCIENCE.

### MATERIA MEDICA AND THERAPEUTICS.

### MEDICAL SOCIETY OF LONDON.

SATURDAY, DECEMBER 4, 1852.—MR. BISHOP, PRESIDENT.

Dr. DE MERIC showed to the Society the saccharine capsules of copaiba and cubebs, prepared by M. Jozeau, under the name of Copahine-Mège, which have been described in a late number of this journal, (see THE LANCET, vol. ii. 1852, p. 422;) and as this was neither a a secret nor a patented remedy, he was induced to try it in his practice. He stated that he had found them very efficient in the treatment of gonorrhœa, as the mouth did not perceive the taste of the copaiba, and the stomach did not revolt against the drug. Dr. De Méric observed that his patients took the capsules without repugnance, and never complained of any gastric disturbance. He was sure that many of the fellows would find this kind of sugar-plums very convenient in private practice, as they could be carried in the waistcoat-pocket, and taken at leisure. The speaker touched upon several other points, which we need not repeat here, as they may be found in the number of this journal alluded to above.

Mr. CHIPPENDALE wished to know whether the capsules were sold at a high price. He asked the question because he might be inclined to use them in his dispensary.

Dr. DE MERIC was not prepared to give the information required, as he had confined himself to the pharmaceutical bearing of the matter. But he might venture to say that M. Jozeau would probably charge hospitals and dispensaries a very low price. He thought, at the same time, that the capsules were more calculated for private than public practice.

The PRESIDENT inquired the actual amount of copaiba and cubebs in each capsule.

Dr. DE MERIC could not bring to mind the exact quantity; but we have since been informed that it is sixteen grains of prepared copaiba and about two grains of cubebs for each capsule.

